# Recent *Mycobacterium tuberculosis* Transmission, United States, 2018–2022 (Response)

**DOI:** 10.3201/eid3209.261478

**Published:** 2026-09

**Authors:** Steve Kammerer

**Affiliations:** Centers for Disease Control and Prevention, Atlanta, Georgia, USA

**Keywords:** tuberculosis and other mycobacteria, respiratory infections, *Mycobacterium tuberculosis*, transmission, whole-genome sequencing, machine learning, diagnostics, bacteria, United States

**In Response:** We thank Dr. Zubairi for raising the points in his letter (1). We view those methodological considerations as valid questions within the context of applying our results to tuberculosis (TB) prevention and control.

We agree that transmission networks can be more complex than are represented by our plausible source case algorithm and use of whole-genome single nucleotide polymorphism distances to assess genomic similarity. However, our integration of spatial and temporal proximity with genomic similarity represents our best estimates of recent transmission, which were validated with locally provided contact investigation data from multiple sites in the United States. In addition, we accept that those estimates can misclassify cases as false-positive or false-negative transmission events. Validation of the algorithm demonstrated an accuracy of 95.8% compared with on-the-ground epidemiologic data, with sensitivity of 79.1% and specificity of 97.7% (S. Kammerer, unpub. data)

Regarding external validation of the adaptive boosting machine learning model, our study examined the epidemiology of TB transmission in the United States. Generalization to other settings with different transmission dynamics, demographics, genomic diversity, healthcare utilization, and public health infrastructure, including settings that do not genotype nearly all isolates from culture-confirmed cases, would not be appropriate. Our findings are appropriate to consider for prioritizing interventions in the United States. The methodological approach could also be a starting place for a similar analysis in other settings with similar epidemiology and genotyping coverage.

We agree that diagnostic delay is a major driver of TB transmission; however, we did not have reliable data regarding symptom onset to assess in our study. Accurate data regarding symptom onset are challenging to obtain in clinical settings. However, we did consider objective clinical findings associated with diagnostic delay and advanced disease (e.g., sputum smear positivity), and those findings were associated with plausible source cases in our analysis.
